# “The big win was that we actually cooked”– a qualitative interview study about the parental experience of Family Meals on Prescription for children living with obesity

**DOI:** 10.1186/s41043-025-00921-3

**Published:** 2025-05-24

**Authors:** Terese Torstensson, Gerd Almqvist-Tangen, Shawnee Waters, Jenny M. Kindblom, Josefine Roswall, John E. Chaplin, Lovisa Sjogren

**Affiliations:** 1 Department of Pediatrics, Institute of Clinical Sciences, Sahlgrenska Academy University of Gothenburg, Gothenburg, Sweden; 2 Department of Pediatrics, Hallands Hospital Halmstad, Halmstad, Sweden; 3 Department of Pediatrics, Region Västra Götaland, Sahlgrenska University Hospital, Gothenburg, Sweden; 4 Institute of Medicine, Sahlgrenska Academy at University of Gothenburg, Gothenburg, Sweden; 5 Department of Drug Treatment, Sahlgrenska University Hospital, Region Västra Götaland, Gothenburg, Sweden

**Keywords:** (1–7) Childhood obesity, Dietary intervention, Family experience, Family meals

## Abstract

**Purpose:**

The aim was to evaluate the parental experiences of participating in the randomized controlled trial “Family Meals on Prescription” for childhood obesity. The intervention consisted of a weekly prepacked grocery bag with recipes to cover five evening meals for the whole family.

**Methods:**

Parental, semi-structed interviews were performed with at least one parent of the participating families. Inclusion criteria was that the family had actively participated in the intervention. The interview questions were formulated to explore the experiences, opinions and attitudes of the parents regarding the intervention. The interviews were transcribed and thematically analysed using both an inductive and deductive approach.

**Results:**

Parents (six mothers and five fathers) from ten families were interviewed. The thematic analysis identified that the intervention reduced stress and was supportive regarding meal planning. The parents described an increased knowledge concerning food and cooking in the whole family and changes in meal behaviours such as more regular meals, trying new food and consuming more vegetables. Negative experience concerning the portion size being too large making it hard to maintain boundaries were also highlighted.

**Conclusion:**

Parents described that the intervention with a subsidized prepacked grocery bag supported them in adapting more positive behaviours related to family meals through decreased stress levels and including the children in the daily cooking in an encouraging way. This method has the potential to help this group by directly engaging the whole families in new behaviours and enhancing family cohesion.

**Registered:**

Registered at clinicaltrals.gov 27 Nov 2020, retrospectively registered: clinicaltrials.gov number 19,002,468. https://clinicaltrials.gov/study/NCT05225350.

**What is Known:**

Regular family meals and mealtime routines has been shown to be important for nutritional health and dietary patterns in children and adolescents. Intensive dietary intervention with a participatory approach in the home setting may be a novel tool in the treatment of childhood obesity.

**What is New:**

This study describes the parental experience of taking part of an intensive dietary intervention consisting of receiving prepacked grocery bags with the purpose to promote family meals. The parents described that the intervention led to more positive behaviours related to family meals.

**Supplementary Information:**

The online version contains supplementary material available at 10.1186/s41043-025-00921-3.

## Background

In Sweden the prevalence of obesity among children and adolescents is estimated to between 4 and 9% [1, 2]. This makes obesity one of the most common chronic diseases in children, and once established, it often persists into adulthood [3–5]. Evidence shows that behavioural and lifestyle treatment from an early age is more successful than treatment initiated in adolescence [6]. Further, there is evidence that successful treatment decreases the risk of mortality and severe comorbidities [7]. This knowledge motivates treatment start at the youngest age possible to obtain the best results [8].

Family lifestyle intervention programmes are acknowledged as a key component in the treatment of paediatric obesity and should include support to behavioural change regarding diet, physical activity, and sleep [9–11]. There is an increasing interest in interventions delivered in the home setting involving the whole family [10, 12].

Many factors affect eating habits among children and adolescents, their parents, and the family´s routines, such as, which foods are accessible in the home environment and which meals are regularly prepared [13]. Adolescents’ state that taste is the primary factor influencing food choice followed by familiarity, which is often based on their own family´s habits [14, 15]. Among younger children, parents have an even larger effect on the children´s eating habits and weight development [16]. The parent is a role model for their child and has the power to choose what food to cook and serve for their family, underlining the importance of involving the whole family in dietary changes included in the treatment of childhood obesity [15].

Shared family meals are an important occasion where children can learn from their parents and develop long-lasting eating habits [17]. Studies reveal that shared family meals tend to be connected to a healthier diet which includes more vegetables and fruits and less sweetened beverages and fast food [17–20]. Furthermore, it has also been shown that more frequent family meals are associated with a healthy diet and the risk of developing overweight and obesity is lower among children who share at least three family meals per week [21, 22].

In 2019, a prospective randomized controlled trial named Family Meals on Prescription (FMP) was conducted at two Paediatric outpatient clinics in the Region of Halland [23]. The study explored an intensive dietary intervention consisting of a subsidized weekly bag of groceries and recipes for five family meals per week for three months, as a novel approach for the treatment of obesity in children and adolescents. The intention was to provide a method to stimulate the whole family to participate in the food preparation. Therefore, meals had to be varied, interesting and easy to prepare.

## Aim

The aim of this study was to evaluate parental experiences and opinions of participating in the Family Meals on Prescription (FMP) study.

The specific research questions were: How have the parents experienced the intervention? Has participation in the study led to any perceived life-style changes within the families?

## Methods

The study procedure of the FMP has been described in detail elsewhere [23]. In short, all families with children aged 5 to 15 attending the obesity treatment program were invited to an information meeting regarding the intervention study. The patient families were also informed during visits at the clinic. The intervention was designed to encourage a participatory approach to strengthen the possibility of sustained dietary change, by the families delivering the intervention themselves [23–25]. The FMP was approved by the regional ethical board in Lund (reference number 2018/836) and ethical principles for medical research were followed (Helsinki declaration). The study has been registered in ClinicalTrials.gov (NCT05225350).

The inclusion criteria for families to join the present interview study were having a child or adolescent participating in the FMP and understanding and speaking Swedish. The parents were asked by their physician to participate in the interviews during their child’s annual follow up and all families were asked consecutively. The participants were given written and oral information regarding the study protocol and were given the opportunity to ask questions regarding the study. Upon return of the informed consent form the family was contacted by the interviewer (SW) to arrange the time and place of the interview. All parents who received information regarding the interview study decided to participate.

Qualitative semi-structured interviews with the parents alone were performed in order to explore relevant issues and identify expectations, experience, opinions and attitudes towards using the grocery bag, as well as how these opinions were formed and sustained [26]. Exploratory questions (supplement 1) were formulated by the research team based on clinical experience and review of the research. One pilot interview was performed to assess participant comprehension, timing, practice a non-judgemental interviewing technique and to secure that the questions addressed the objective of the study. The interview guide and analysis process were refined following preliminary analysis of the first interviews to allow greater focus on the research questions. Interviewing continued until SW and JC judged that no new information was forthcoming, and saturation was reached.

The interviews were conducted, recorded by audio and transcribed verbatim by author SW, who had no prior relations with any of the participants. SW was a last semester medical student when the interviews were conducted, and she was supervised by JC with a long experience in the field of qualitative research. The qualitative data was assessed using thematic analysis [27]. A reason action approach to the analysis was used to identify, interpret and report relevant patterns within the data. This technique used a combination of inductive and deductive reasoning to explore, interpret and uncover themes related to a research question [24]. The transcripts were transferred into the software program NVivo 11. The codes were labelled with short descriptions of the content and then used to generate broader themes, clarifying the patterns found in the transcripts (SW and JC), see Table 1. Throughout the process, the coding and characterisation of themes, was discussed with members of the research group who has a vast experience in the field (JC and GA-T). LS and TT subsequently reflected on the subcategories and categories and revised them until consensus was reached, in order to enhance trustworthiness.

**Table 1:** Example of the coding process and creating themes

Quote	Code	Theme
That she [the child] got it. She got chosen, that was good.	Positive experience and attitude	Building a family attitude towards everyday family meals
Yes. That it becomes one of those things you can do together, like, not only eating together, but also cook the food together. The evening meal became a little more of a happening, or so	Create a positive collaboration	
No, but for us it wasn´t the ultimate experience, since the problem is that she [the child] couldn’t eat… for her it is hard to eat casseroles and that.	Groceries and recipes the family didn’t like	
We probably do the shopping as usual, but more vegetables after having the prepacked grocery bags. Now it’s not possible, it’s not possible to have a dinner without a salad. We all eat salad. And before… you got to try quite a lot of new vegetables when we had the grocery bag.	Started to eat more vegetables	Awareness of behavioural changes
Yes, it works, you have discovered new things you didn’t know about. And things we cooked before, maybe with cream, then you used something else instead of the cream, and it became precisely as tasty.	New knowledge about cooking	
And that we cook together, that is also a win, since it was made for that, that we could… everyone could participate. That we got used to, that was also good.	Starting to cook together, including the children	
People are looking at you and “well, she is just at home eating unhealthy stuff”, and then you are struggling and toiling with eating right and be as physically active as possible, and that you could manage and still you get the preconceived notions. And that is what make you perhaps unable to keep on going. You think like, now I quit, I can’t stand it anymore	Others negative attitudes/norms decreasing the motivation	Awareness of key factors that lead to change
The planning, to get variation and for us the grocery bag had been that. Yes. It is hard otherwise, with time and… yes. Everyone is tired and hungry when you are coming home…	Stress and lack of time has a negative influence on habits	
But at the weekend if you decide together, what or if someone wants something special for the week, and yes. We talk together. Then I also believe the best thing would be if you sat down and did a weekly menu of what to eat every day, so that is… decided.	Hard to plan by yourself, it becomes better if you make a plan	

## Results

Of the 48 families participating in the intervention parents of ten families (21%) participated in the present interview study. See family characteristics in Table 2. The first four interviews were conducted in person and the six interviews that followed were conducted over the telephone due to COVID-19 recommendations. Interviews lasted between 17 and 37 min.

**Table 2:** Participants characteristics

Interview	Interviewee	Household	Children in obesity treatment
1	Father	1 adult, 1 child	1
2	Mother	1 adult, 2 children	2
3	Father	2 adults, 3 children	1
4	Mother	2 adults, 2 children	1
5	Mother	2 adults, 2 children	2
6	Mother	2 adults, 5 children	1
7	Mother	2 adults, 2 children	1
8	Mother, Father	2 adults, 1 child	1
9	Father	2 adults, 2 children	1
10	Father	2 adults, 2 children	1

Three major themes were identified:

1. Building a family attitude towards everyday family meals.
2. Awareness of behavioural changes.
3. Awareness of key factors that lead to change.

### 1) Building a family attitude towards everyday family meals

The expectations parents had of participating in the study varied. The parents mentioned wanting to learn new recipes and find inspiration to cook again. Parents also mentioned that they had hoped their child would stabilise in weight or even lose weight, while others said that they did not care if their child lost weight or not, and that their primary goal was to create healthy eating habits.

Parents spoke about feeling privileged when they each week collected their weekly grocery bag. The parents expressed that stress and lack of time makes it difficult to plan and cook properly, or in the way that they wished to do, but the grocery bags lifted some of this stress and took pressure off planning and preparing meals. The subsidized bag served as a financial support as well. Having the selection of meals and menus prepared by someone else was described as both exciting, however mainly a relief. This relief from stress helped create a more positive atmosphere around family dinners, instead of tension and unease.

*”M2: I am a single mother with two kids*,* I have quite a lot to do anyway*,* and if I wouldn’t have to make all these decisions*,* because there are a lot of decisions*,* I mean every time you go grocery shopping*,* there are lots of decisions*,* lots of things to keep in mind*,* lots to read. If I didn’t have to do that […] then I could relax.*

*“P3: Or*,* that’s where the stress comes in*,* I mean*,* the parental stress*,* like*,* “oh my god*,* what are we going to cook today*,* oh god I don’t know what to make”. And then you often put that pressure on a child [to decide what to eat]. […] “Okay kids*,* what are we having today?” […] It is so damn easy it takes that direction*,* and it becomes a kind of negative energy surrounding it. Well*,* it is very nice to have it [the grocery bag].”*

Another aspect the parents spoke of as positive was that using the prepacked grocery bags made it easier to involve the children in cooking. It became a nice event to collect the bags, look through the recipes or prepare and eat the meals together.

*“P8: Yes. That it becomes one of those things you can do together*,* like*,* not only eating together*,* but also cook the food together. The evening meal became a little more of a happening*,* or so"*.

Discovering new recipes and groceries made it enjoyable to cook the meals. The parents described that during the intervention they realised that cooking from scratch did not have to be as difficult as they had thought before and said that they re-discovered their knowledge regarding cooking.

*”M4: like*,* plaice fillets and bread them yourself and then fry them. It takes no time*,* but you never think about it. […] It isn’t like*,* really hard. Maybe one can believe that*,* before you are forced to do it with the bag [and the prepacked recipes].”*

*”M7: And*,* like I said*,* the recipes are simple*,* and it’s actually true that it doesn’t take much time*,* even if you from the start felt that “Oh*,* well this felt…”*,* maybe complicated? But then no*,* it was easy. It doesn’t take much time.”*

The parents mention some aspects of the intervention that was highlighted as obstacles to promote shared family meals. The following aspects were mentioned; sometimes the families did not enjoy the recipes or groceries, and it could be difficult to convince the children to try and taste the new foods. They also mentioned that some did not consider the food in the grocery bag to be healthy enough, referring to prefabricated food, products with a high fat percentage or meals containing carbohydrates. The parents also wished for more fruit and vegetables. Some parents thought the portion sizes were too big, making it difficult for them to set boundaries for their child, for example when the child saw that there was food left-over, and the parents tried to tell them that everybody only receives one plate each. This could lead to tension or conflict.

M4: *Well, now I love food, and it doesn’t bother me if there are leftovers for the next day, but it can be difficult to explain to the child, if it is a child that it is about, that there are eight pieces of fish there, but we are not eating all of those eight fishes now. It is pretty difficult math. […] or I struggle with that communication, “no, you can’t have more”, right? It becomes such a negative atmosphere?*

Parents also expressed that they could feel somewhat limited by the frequency of food, sometimes there was even food left during the weekends, when they might have wished to cook something different. Some of the families wanted more freedom or flexibility in their cooking. It could also be tiring to constantly have new dishes and new types of food. The parents expressed that receiving meals for three days would be preferable to five.

*”M4: So*,* since there was so much food*,* and I don’t throw away any leftovers*,* I save them for the next day*,* and then you are supposed to cook something new again?”*

### 2) Awareness of behavioural changes

The parents spoke about having learned new recipes and discovered new groceries that the families enjoyed, and that these new dishes had been incorporated in their cooking after the study was completed. Since the recipes were straight forward to cook, they also said that it was easier to involve their children in the preparation and cooking of meals. This led to behavioural changes in the family engaging the children in cooking, some children could start to cook the recipes on their own, while others helped with for example preparations.

*”M7: We have saved the booklets we were handed with the recipes*,* and then sometimes we say*,* “oh*,* that thing was so tasty*,* should we cook it?” So we still use it sometimes.”*

*”P10: So the kids can*,* like*,* start prepping the food and they can participate in the cooking*,* and they sort of think it is fun to be a part of it and doing it too. Feeling involved in it.”*

The parents who perceived the portion sizes as adequate spoke about how it had helped them learn how much food to cook after the intervention, making it easier to estimate appropriate portions. For example, they described how they change their behavioural and now sometimes weighed the ingredients to see how much they would need for a certain number of portions.

*”P1: I know that I have made all of these recipes from the grocery bags*,* and then I’ve noticed that four servings*,* that’s about 600 g of chicken. When I make chicken some other time*,* I have that in the back of my mind*,* that okay*,* 600 g was for four people*,* so then I use the scale to measure that. Otherwise*,* I would just have thrown in the entire bag. So that’s great.”*

The parents expressed that it can be difficult to try to motivate their children to taste new foods. During the intervention they experienced that their children got used to having new dishes almost every day, and this led to their children learning to at least taste the food to a greater extent than before. This lasted after the study was completed, making it easier to introduce new foods and make changes in their dietary habits, and helped create an interest in cooking and food among the children as well.

*“M7: they got used to it [new foods]*,* and said themselves that “oh”*,* and they can still say it*,* “that thing we had was nice*,* can’t we make it again”. So*,* from being a little bit like it was weird food from the beginning*,* their attitude changed too? The attitude*,* after a while.”*

Receiving new recipes every week during the intervention also resulted in a change for families who began to search for new recipes on their own, outside of the study. The parents said that usually they tried to cook something after the children came home from school, but that this was not always the case. Using the grocery bag became a motivation, according to the parents, to cook dinners every day, resulting in more regular mealtimes and lasting satiety.

*”M2: The big win was that we actually cooked. Which we cheat with quite wildly when we don’t have the grocery bag.”*

*M6:* *And then we’ve had… Yes, now she’s eating more regularly, so now we feel that maybe things can turn around. But it takes time.*

In addition to trying new recipes, the parents further spoke of learning new ways to cook that they perceived as healthier. For example, they said that they had changed to using oil instead of butter, low-fat dairy products and eating more vegetables than before.

*”P9: We probably do the shopping as usual*,* but have added more vegetables after having the prepacked grocery bags. Now it’s not possible*,* it’s not possible to have a dinner without a salad. We all eat salad. And before… you got to try quite a lot of new vegetables when we had the grocery bag.”*

*” P10: So like when you*,* yes*,* when you make root vegetables in the oven. I used to stand there*,* pouring a hell of a lot of oil over them. And*,* like*,* now*,* then they have*,* what is it*,* a tablespoon you should have*,* and then throw it in a bowl and mix it in the bowl. “*.

### 3) Awareness of key factors that lead to change

The parents highlighted that many different factors influenced their decision making and the way they behaved regarding eating habits, both during the intervention and after its completion. There were general aspects, but also things directly concerning the intervention. Parents also had ideas about what kind of support or help they wanted when it comes to lifestyle changes.

Parents stressed the need for an intention, or a willingness, to try and change the behaviour for it to work. It was also seen as very important to have the right knowledge about matters such as which foods are nutritious, healthy recipes, how to involve the children in the daily routines regarding mealtime preparations and food, and how to set boundaries in a good way.

*”P8: I think it is important that you are interested*,* open and interested in changing your*,* your way of eating. I mean what you eat and how you eat*,* how you cook together*,* if you eat together.”*

The parents spoke of the financial aspect, if they had not been in the study, some might not be able to afford the weekly grocery bags at the regular price. One thing that strongly influenced what groceries they normally purchased in the stores was the price, and many often-bought foods on special offers. The parents said that they tried different types of food during the study than they would not have bought otherwise. Not being able to buy groceries that were considered healthy due to high costs was perceived as a barrier towards changing behaviour and food habits.

*P9: We would have liked to have continued with it [the prepacked grocery bag] but… My finances do not actually allow it.*

Another factor that could impede attempts at lifestyle changes was the level of stress among the parents. Stress rendered it difficult to properly plan dinners, and during the weekdays there was normally not enough time to shop, prepare and cook family meals. The situation around mealtimes could also be stressful, the children can be picky and there might be quarrels about what to eat. A negative atmosphere surrounding food adds to the stress level, further hindering attempts to change behaviours within the family. In addition, to the parents being stressed, they said that stress or negative thoughts about themselves among the children had a very negative effect on their motivation. Parents worried that overemphasis on weight management and healthy eating might be harmful for their children.

*“P3: There was a lot of that [shopping food] on the way home from work*,* some major grocery shopping every now and then*,* but there is always this god damn anxiety when you have to choose what to cook*,* a lot of opinions. Three children. Everyone wants something different.”*

## Discussion

In this study, parents were interviewed about their experiences of a participatory, intensive dietary intervention, as a part of the treatment of obesity among children and adolescents. The interviews identified three major themes: (1) building a family attitude towards everyday family meals, (2) awareness of behavioural changes, (3) awareness of key factors that lead to change.

The parents spoke of the challenges of trying to plan, prepare and cook healthy meals for the families during weekdays. They expressed that stress and lack of time and ideas about what to serve created a negative atmosphere, making it difficult to try and change eating habits. However, during the Family Meals on Prescription intervention, the parents described how some of this pressure lifted.

Parents expressed that they were experiencing a great deal of stress. They described being under constant pressure and not finding the time to plan or properly prepare meals as a cause of negative emotions and a complicating factor when it came to making behavioural changes. This stress may favour meal decision that the parent may regret [28]. Stress is related to poorer food choices and higher intake of unhealthy food [29]. Even though the parents knew that planning meals beforehand made it easier to make healthy choices, they still found this very difficult, referring to stress and lack of necessary skills. It has been shown that a higher stress level is associated with less healthy food, servings of vegetable at dinner in a lower degree, and more frequently serving fast food [18]. The parents described that receiving the grocery bags saved time and removed the need of the parents to plan the dinners beforehand. However, it could be beneficial for the parents to receive support that could provide them with the necessary skills to do this on their own. Previous research investigating childhood obesity treatment shows that changing behaviour and lifestyle is difficult [6, 8]. Parents greatly influence the food habits of their children, yet still many emphasize the responsibilities of the schools to help create healthy eating behaviours among the children [30]. Previous studies have also found that many parents consider their children to be responsible for their current eating habits and thereby also for changing their behaviour [31, 32]. Higher conflict levels in families have been associated with higher BMI among the children [33–35]. Importantly, conflict levels within families have also been linked with eating patterns associated with overweight and obesity [33]. However, interventions aimed at integrating the parents in the treatment of childhood obesity have led to changes in these attitudes [31]. Significant associations have been found linking positive family- and parent-level food-related dynamics and reduced risk of childhood obesity [22]. Interventions focused on behavioural patterns of entire families as well as family dynamics therefore has great significance in the treatment of paediatric obesity. Several barriers and facilitators of behavioural change were identified through the interviews, such as, lack of time, stress within the families, lack of knowledge about healthy food, financial constraints, an unwillingness to try new foods and difficulties in setting boundaries.

The parents experienced that the intervention led to convenient time saving, enabling the parents to focus their attention on for example cooking instead of planning or coming up with ideas about what to make for dinner, thus reducing stress. The parents in this study expressed that stress within the families, especially regarding food, lead to quarrelling and a negative atmosphere surrounding mealtimes. This negativity in connection to meals could enhance feelings of unease concerning food that already exists in this group, further hindering attempts at positive changes.

Another barrier toward making healthy choices that parents described was economic limitations, this and not having enough time to cook have been reported from previous studies [28, 30]. The economic aspect was mentioned as the main reason why parents did not use prepacked grocery bags to facilitate everyday life by themselves. This underlines the importance of financial support when offering lifestyle treatment with an enhanced dietary component.

The parents spoke about primarily wanting help with how to plan meals, expressed a wish to learn new recipes and gain knowledge about healthy food. They highlighted that the children influenced what kind of food they would cook at home. The children were often particular about which foods they liked and had a negative attitude toward trying new things. Reducing fussy eating has been identified as an important component to achieve a healthier diet among children with obesity [36]. To expand children’s taste preferences, parents are recommended to repeatedly serve different foods [37]. For some children accepting new tastes can be very demanding. A previous study showed that parents sometimes avoid new foods in order to minimize conflicts regarding the meals that are served [28]. In this study, parents experienced that their children gradually started to taste more varied foods after a few weeks of using the prepacked grocery bag.

The parents also reported that they had begun to eat more vegetables within the families after the intervention. These positive changes can be seen as ways to promote healthy eating behaviours among children. Since eating behaviours are influenced by friends and family, school environment and the availability of different foods, it is important to make it easier to choose healthy options [30]. Importantly, families in this study reported that the intervention increased the accessibility to vegetables. Further, the parents spoke of learning or rediscovering that cooking meals from scratch did not have to be too complicated.

Involving the children also had a positive effect on the atmosphere in the family’s concerning food. Creating an interest for preparing and cooking meals among the children was perceived as encouraging. This could lead to greater confidence among children and parents, thereby increasing self-efficacy and motivation, which further strengthens intention toward change [12, 38]. This study showed that parents appreciated involving the children in mealtime preparations and cooking as well as planning the meals.

Parents participating in the study mentioned an apprehension regarding too much focus on weight management or healthy eating could be harmful or have a negative effect on their children. Similar findings have been shown in previous research, where parents have reported that they experience an inner conflict between on the one hand wanting their child to have a strong self-confidence, while at the same time trying to help them lose weight [39]. During this intervention, the parents experienced an increased level of cohesion regarding food within the families. Focussing on the positive aspects of doing something together as a family instead of the negative aspects of weight management has the possibility to promote healthy eating without enhancing negative emotions regarding self-worth among the children.

There were also negative experiences regarding the intervention, the most pronounced being the large portion sizes which could make it difficult to set boundaries and possibly leading to increased stress or conflict in the families. On the other hand, some parents appreciated that the grocery bags were useful when it came to estimating portion sizes. In this trial the grocery bags were similar for all families regardless of the age of the children, which might explain the heterogenous responds regarding the portion sizes. All families in the present study had received age adjusted Meal Sizer^®^ (a portion control tool) but still found setting boundaries regarding portion size difficult. This finding is in accordance with previous studies describing that parents wish for support regarding limiting portion size [26, 27]. The implementation of new rules regarding food has been reported as challenging in several studies. Parents experience difficulties with upholding boundaries within the families, as well as in other social contexts such as in school, at parties or among the extended family [26, 28]. Receiving help with properly estimating portion sizes has the potential to be very helpful, but if this method is to be used in similar interventions in the future, more support concerning parental enforcement of the portion size to make sure it does not have the opposite effect might be of value.

However, there are possible ways in which the method could be improved in the future. If this intervention were to be carried out at a larger scale, the recipes should be more optimized for this patient group and more support for limiting the portion sizes need to be given. Some parents suggested that three meals per week would be more feasible and also wished for additional healthy snacks.

This study provides a deeper understanding of the FMP intervention effects from the families’ perspective, and insights into possible ways to improve future applications of the method.

### Strengths and limitations

A strength of this study was a sample of participants that came from a varied population with different duration of obesity treatment, children of different ages, and families that lived in different cities [23]. Another strength of this study was the multi-professional scientific team with both female and male researchers with a long experience in the field. TT is a paediatric dietitian and JK, JR and LS are medical doctors, all active in the paediatric obesity field. GA-T is a paediatric nurse, SW was a last year medical student at the time of this study, and JC is phycologist.

One limitation of this study was that the interviews were conducted in different manors (e.g. in person or over the phone) due to COVID-19 pandemic. Another limitation is the lack of data regarding social determinants of health.

## Conclusions

This study investigated the parental experience of participating in the Family Meals on Prescription intervention. Parents reported developing more positive attitudes regarding family meals, highlighted that behavioural change was possible and identified key factors that had prevented change in the past. The parents reported several perceived behavioural changes after participating in the project, such as planning and cooking meals together, introducing new foods and eating more vegetables. This study shows that parents appreciated the intervention in the FMP study that investigated a participatory approach involving the whole family in the home setting.

## Electronic supplementary material

Below is the link to the electronic supplementary material.

Supplementary Material 1

## Data Availability

No datasets were generated or analysed during the current study.

## Abbrevations

FMP: Family Meals on Prescription

## References

[1] Bygdell M, Célind J, Lilja L, Martikainen J, Simonson L, Sjögren L, et al. Prevalence of overweight and obesity from 5 to 19 years of age in Gothenburg, Sweden. Acta Paediatr. 2021;110(12):3349–55. 34464992 10.1111/apa.16089

[2] Eriksson M, Lingfors H, Golsäter M. Trends in prevalence of thinness, overweight and obesity among Swedish children and adolescents between 2004 and 2015. Acta Paediatr. 2018;107(10):1818–25. 29637596 10.1111/apa.14356

[3] Geserick M, Vogel M, Gausche R, Lipek T, Spielau U, Keller E, et al. Acceleration of BMI in early childhood and risk of sustained obesity. N Engl J Med. 2018;379(14):1303–12. 30281992 10.1056/NEJMoa1803527

[4] Ward ZJ, Long MW, Resch SC, Giles CM, Cradock AL, Gortmaker SL. Simulation of growth trajectories of childhood obesity into adulthood. N Engl J Med. 2017;377(22):2145–53. 29171811 10.1056/NEJMoa1703860 PMC9036858

[5] Marcus C, Danielsson P, Hagman E. Pediatric obesity-Long-term consequences and effect of weight loss. J Intern Med. 2022;292(6):870–91. 35883220 10.1111/joim.13547 PMC9805112

[6] Danielsson P, Kowalski J, Ekblom Ö, Marcus C. Response of severely obese children and adolescents to behavioral treatment. Arch Pediatr Adolesc Med. 2012;166(12):1103–8. 23108856 10.1001/2013.jamapediatrics.319

[7] Putri RR, Danielsson P, Ekström N, Ericsson Å, Lindberg L, Marcus C et al. Effect of pediatric obesity treatment on Long-Term health. JAMA Pediatr. 2025;179(3):302–309 10.1001/jamapediatrics.2024.5552 PMC11877215 39836390

[8] Danielsson P, Svensson V, Kowalski J, Nyberg G, Ekblom O, Marcus C. Importance of age for 3-year continuous behavioral obesity treatment success and dropout rate. Obes Facts. 2012;5(1):34–44. 22433615 10.1159/000336060

[9] Duncanson KS, Collins VA, The DiET-CO consortium, Obesity for the World Health Organization. Prepared for the World Health Organization Priority Research Centre in Physical Activity and Nutrition, School of Health Sciences, Faculty of Health and Medicine, University of Newcastle. CE and. Interim Report on the Effectiveness of Dietary Interventions for Children and Adolescents with Overweight and 2017 December 2017. Contract No.: ISBN 978-0-7259-0017-5.

[10] Hampl SE, Hassink SG, Skinner AC, Armstrong SC, Barlow SE, Bolling CF et al. Clinical practice guideline for the evaluation and treatment of children and adolescents with obesity. Pediatrics. 2023 Feb 1;151(2):e2022060640. 10.1542/peds.2022-060640 36622115

[11] Sundbom M, Järvholm K, Sjögren L, Nowicka P, Lagerros YT. Obesity treatment in adolescents and adults in the era of personalized medicine. J Intern Med. 2024;296(2):139–55. 39007440 10.1111/joim.13816

[12] Appelhans BM, Moss OA, Cerwinske LA. Systematic review of paediatric weight management interventions delivered in the home setting. Obes Rev. 2016;17(10):977–88. 27231126 10.1111/obr.12427

[13] Roblin L. Childhood obesity: food, nutrient, and eating-habit trends and influences. Appl Physiol Nutr Metab. 2007;32(4):635–45. 17622277 10.1139/H07-046

[14] Contento IR, Williams SS, Michela JL, Franklin AB. Understanding the food choice process of adolescents in the context of family and friends. J Adolesc Health. 2006;38(5):575–82. 16635770 10.1016/j.jadohealth.2005.05.025

[15] Koivisto Hursti UK. Factors influencing children’s food choice. Ann Med. 1999;31(Suppl 1):26–32. 10342497

[16] Birch LL, Davison KK. Family environmental factors influencing the developing behavioral controls of food intake and childhood overweight. Pediatr Clin North Am. 2001;48(4):893–907. 11494642 10.1016/s0031-3955(05)70347-3

[17] Gillman MW, Rifas-Shiman SL, Frazier AL, Rockett HR, Camargo CA Jr., Field AE, et al. Family dinner and diet quality among older children and adolescents. Arch Fam Med. 2000;9(3):235–40. 10728109 10.1001/archfami.9.3.235

[18] Neumark-Sztainer D, Hannan PJ, Story M, Croll J, Perry C. Family meal patterns: associations with sociodemographic characteristics and improved dietary intake among adolescents. J Am Diet Assoc. 2003;103(3):317–22. 12616252 10.1053/jada.2003.50048

[19] Taveras EM, Rifas-Shiman SL, Berkey CS, Rockett HR, Field AE, Frazier AL, et al. Family dinner and adolescent overweight. Obes Res. 2005;13(5):900–6. 15919844 10.1038/oby.2005.104

[20] Walton K, Horton NJ, Rifas-Shiman SL, Field AE, Austin SB, Haycraft E, et al. Exploring the role of family functioning in the association between frequency of family dinners and dietary intake among adolescents and young adults. JAMA Netw Open. 2018;1(7):e185217. 30646382 10.1001/jamanetworkopen.2018.5217 PMC6324390

[21] Veugelers PJ, Fitzgerald AL. Prevalence of and risk factors for childhood overweight and obesity. CMAJ. 2005;173(6):607–13. 16157724 10.1503/cmaj.050445 PMC1197160

[22] Berge JM, Rowley S, Trofholz A, Hanson C, Rueter M, MacLehose RF, et al. Childhood obesity and interpersonal dynamics during family meals. Pediatrics. 2014;134(5):923–32. 25311603 10.1542/peds.2014-1936 PMC4210801

[23] Torstensson T, Bohlin A, Almqvist-Tangen G, Roswall J, Kindblom JM, Sjogren L. Family meals on prescription as treatment for childhood obesity-a randomized controlled trial. Eur J Pediatr. 2024;183(11):4857–486. 10.1007/s00431-024-05744-8 PMC11473609 39251447

[24] Bergold J, Thomas S. Participatory research methods: A methodological approach in motion. Hist Social Research/Historische Sozialforschung. 2012;13(1):191–222.

[25] Cornwall A, Jewkes R. What is participatory research? Soc Sci Med. 1995;41(12):1667–76. 8746866 10.1016/0277-9536(95)00127-s

[26] Doody O, Noonan M. Preparing and conducting interviews to collect data. Nurse Res. 2013;20(5):28–32. 23687846 10.7748/nr2013.05.20.5.28.e327

[27] Braun V, Clarke V. Using thematic analysis in psychology. Qualitative Res Psychol. 2006;3(2):77–101.

[28] Dahl AA, Mayfield M, Fernandez-Borunda A, Butts SJ, Grafals M, Racine EF. Dinner planning and Preparation considerations of parents with children attending childcare. Appetite. 2023;180:106332. 36202147 10.1016/j.appet.2022.106332

[29] Leeds J, Keith R, Woloshynowych M. Food and mood: exploring the determinants of food choices and the effects of food consumption on mood among women in inner London. World Nutr. 2020;11(1):68–96.

[30] Haerens L, De Bourdeaudhuij I, Barba G, Eiben G, Fernandez J, Hebestreit A, et al. Developing the IDEFICS community-based intervention program to enhance eating behaviors in 2- to 8-year-old children: findings from focus groups with children and parents. Health Educ Res. 2009;24(3):381–93. 18603656 10.1093/her/cyn033

[31] Chamay Weber C, Camparini N, Lanza L, Narring F. Parents’ integration in the treatment of adolescents with obesity: A qualitative study. Fam Syst Health. 2016;34(4):396–403. 27598457 10.1037/fsh0000219

[32] Lindelof A, Nielsen CV, Pedersen BD. Obesity treatment-more than food and exercise: a qualitative study exploring obese adolescents’ and their parents’ views on the former’s obesity. Int J Qual Stud Health Well-being. 2010;5(2):10.3402/qhw.v5i2.5073. 10.3402/qhw.v5i2.5073 PMC2875969 20640019

[33] Darling KE, Ruzicka EB, Fahrenkamp AJ, Sato AF. Perceived stress and obesity-promoting eating behaviors in adolescence: the role of parent-adolescent conflict. Fam Syst Health. 2019;37(1):62–7. 30614721 10.1037/fsh0000387

[34] Hanson CL, Klesges RC, Eck LH, Cigrang JA, Carle DL. Family relations, coping styles, stress, and cardiovascular disease risk factors among children and their parents. Family Syst Med. 1990;8(4):387.

[35] Hooper LM, Burnham JJ, Richey R. Select parent and family system correlates of adolescent current weight status: A pilot study. Family J. 2009;17(1):14–21.

[36] Sandvik P, Kuronen S, Reijs Richards H, Eli K, Ek A, Somaraki M, et al. Associations of preschoolers’ dietary patterns with eating behaviors and parental feeding practices at a 12-month follow-up of obesity treatment. Appetite. 2022;168:105724. 34606942 10.1016/j.appet.2021.105724

[37] Birch LL. Development of food preferences. Annu Rev Nutr. 1999;19:41–62. 10448516 10.1146/annurev.nutr.19.1.41

[38] Fishbein M, Ajzen I. Predicting and changing behavior: the reasoned action approach. Taylor & Francis; 2011.

[39] Andreassen P, Grøn L, Roessler KK. Hiding the plot: parents’ moral dilemmas and strategies when helping their overweight children lose weight. Qual Health Res. 2013;23(10):1333–43. 24019307 10.1177/1049732313505151

