# Supplementary material for: “The big win was that we actually cooked”– a qualitative interview study about the parental experience of Family Meals on Prescription for children living with obesity
Source: J Health Popul Nutr. 2025 May 24;44:171. doi: 10.1186/s41043-025-00921-3 (PMC12102861; doi:10.1186/s41043-025-00921-3)
Supplement: Supplementary file 1 — Supplementary Material 1 [file 41043_2025_921_MOESM1_ESM.docx]

**Supplement 1: Interview guide for the parents participating in the study**

1. Tell us what you thought about being part of the Family Meals on Prescription (FMP) project, what was your experience like?

2. Did the prepacked grocery bag change the way you shop, cook, or eat? In what way?

3. Would you do it again? Why or why not?

4. Would you recommend it to a friend? What would you say are the pros and cons?

5. How did it feel that the groceries and recipes were prepared by someone else?

6. Do you remember what expectations you had when you received the prepacked grocery bag? Tell us.

7. Other thoughts on participating in the project?

8. When it comes to changing how you cook/eat, there may be people who have opinions on what you should or shouldn't do. Who do you think would think it was a good idea to participate or change your eating habits? Why?

9. Who do you think would think it was a bad idea? Why?

10. When we are unsure about what to do, we sometimes look at what others are doing. Who would you look to as an example?

11. Who do you think wouldn’t follow or would have difficulty following dietary advice?

12. What would your friends think of the prepacked grocery bag?

13. What do you think most others would think of the prepacked grocery bag as part of a treatment method?

14. What influences what you/shop/cook/eat today?

15. What do you think would make it easier for you/your family to change your eating habits?

16. What do you think would make it more difficult or hinder you/your family from doing so?

17. What do you think affects how easy or difficult it is to change eating habits?

18. How would you assess the quality of the food?

19. How did you feel during the time you used the prepacked grocery bag?
